# Long-Term Oral Feeding of Lutein-Fortified Milk Increases Voluntary Running Distance in Rats

**DOI:** 10.1371/journal.pone.0093529

**Published:** 2014-04-03

**Authors:** Megumi Matsumoto, Masahito Hagio, Ryo Inoue, Tomohiro Mitani, Masako Yajima, Hiroshi Hara, Takaji Yajima

**Affiliations:** 1 Department of Physical Education, College of Humanities and Sciences, Nihon University, 3-25-40, Sakurajosui, Setagaya-ku, Tokyo, Japan; 2 Research Faculty of Agriculture, Hokkaido University, Kita-9, Nishi-9, Kita-ku, Sapporo, Hokkaido, Japan; 3 Faculty of Life Sciences, Toyo University, 1-1-1 Izumino, Itakura-machi, Ora-gun, Gunma, Japan; 4 Laboratory of Animal Science, Kyoto Prefectural University, Shimogamo, Kyoto, Japan; 5 Graduate School of Medicine, Hokkaido University, Kita-15, Nishi-7, Kita-ku, Sapporo, Hokkaido, Japan; University of Pecs Medical School, Hungary

## Abstract

To evaluate the effects of lutein-fortified milk administration on running exercise, a voluntary wheel-running model was performed in rats. Four-week-old F344 rats were administered test milk (10 mL/kg) daily following a 4-h fasting period, and their running distances were measured each day for a 9-week period. Total weekly running distance significantly increased from the sixth week until the end of the test period in lutein-supplemented rats (lutein-fortified milk administered) compared with control rats (vehicle administered). This increase was not apparent in rats administered lutein alone. In the lutein-fortified-milk exercise group compared with the sedentary control group, carnitine palitroyltransferase 1 (CPT-1), total AMP-activated protein kinase (tAMPK), and phosphorylated AMP-activated protein kinase (pAMPK) contents were significantly increased in the gastrocnemius muscle, with a concomitant decrease in triglyceride and total cholesterol levels in the blood and liver. Furthermore, the lutein level in blood of lutein-administered rats significantly decreased with exercise. These results suggest that lutein-fortified milk may enhance the effect of exercise by effective utilization of lipids when combined with voluntary running.

## Introduction

Metabolic syndrome is a cluster of risk factors of various diseases, including hypertriglyceridemia, hyperglycemia, hypertension, and obesity, which further increase the risk for cardiovascular diseases and diabetes [1]. The pathophysiology of metabolic syndrome is complex, and it has been suggested that increased calorie intake, stress, or low levels of physical activity are key factors leading to the syndrome [2]. To prevent metabolic syndrome, improvement of both dietary and exercise habits is required [3], [4], but, in practice, few people engage in adequate levels of physical activity to maintain health [5]. Moreover, it is difficult for aged or obese people and those with physical injuries to maintain physical activity every day. Thus, it is important to diminish the physical burden or make participation in physical activity easier by maximizing the effect of exercise. Natural compounds that can prevent oxidative stress, such as flavonoids, have been suggested to induce utilization of fatty acids in muscle during exercise, resulting in increased endurance for aerobic activity [6]–[8]. An orally active drug, such as an AMPK agonist, a central control factor of muscle and lipid metabolism, enhances exercise training adaptation and increases endurance without exercise [9]. Thus, oral supplementation with an AMPK agonist during physical activity could lead to enhanced metabolic use of lipids. From these reports, physical activity with proper supplementation is effectively able to utilize lipids, consequently leading to lowered risks of metabolic syndrome and enhanced exercise availability.

In epidemiological studies, the habitual intake of dairy products has been reported to have anti-obesity effects [10], and thus, a higher frequency of dairy product consumption has been demonstrated to lower the risk of metabolic syndrome [11]. Whey protein was found to decrease body fats in human studies and is thought to be a key contributor to anti-obesity effects [12]. Furthermore, milk is an effective delivery vehicle for micronutrients such as lipophilic antioxidants [13] and has the potential to be an effective exercise beverage.

Lutein is a carotenoid pigment that is abundant in spinach and kale [14] and also in the milk of grazing cows [15]. Lutein possesses strong anti-oxidative properties and has suppressive effects on cataracts and carcinogenesis [16], [17]. Furthermore, it has been suggested that successive consumption of spinach, a major source of lutein, ameliorates age-related deficits in a rat model of aged-related cognitive decline [18]. Lutein intake with dairy products would be anticipated to enhance its absorption due to its lipophilicity [13]. Furthermore, the fortification of frequently consumed foods has been demonstrated to be an effective and low-cost way to increase body micronutrient supplies and to reduce the incidence of antioxidant deficiencies [19].

A paucity of information exists regarding the physiological impact of cow milk on muscle metabolism during exercise. Furthermore, the effect of lutein intake on muscle metabolism and the effect of its combination with milk are not well known. In this study, we sought to investigate the effects of long-term oral feeding of cow milk supplemented with lutein (lutein-fortified milk) on muscle metabolism using voluntary wheel running in rats.

## Materials and Methods

### Chemicals

Marigold pigment (containing 20% lutein) was kindly provided by Miyako Kagaku Co., Ltd. (Tokyo, Japan).

### Animals and Diet

Male 3-week-old F344 rats (Japan SLC, Shizuoka, Japan) weighing approximately 50 g were individually housed in stainless steel cages with wire-mesh bottoms. The cages were placed in a room with a controlled temperature (22–24°C), relative humidity (40–60%), and lighting (lights on 05∶00–17∶00 h). The rats were allowed free access to deionized water and a semi-purified diet based on the AIN93G formulation [20] throughout the acclimation and experimental periods. The acclimation period was 7 days [21]. After the first 3 days, rats were assigned by matched body weight to either a sedentary group or a voluntary wheel-running (exercise) group containing 24 rats. During the next 4 days, rats in the sedentary group remained in standard wire-bottomed cages, whereas the rats in the exercise group were moved to cages equipped with a running wheel to which they had free access (CLEA Japan, Inc., Tokyo, Japan; diameter, 31.8 cm; width, 10 cm). Running distances were measured using a rotation counter on the cages and recorded regardless of direction (SN-451-B, Sinanoseisakusho, Tokyo, Japan).

The Hokkaido University Animal Committee approved this study, and all animals were maintained in accordance with the Hokkaido University guidelines for the care and use of laboratory animals.

### Preparation of Test Milk

Commercially available cow milk was obtained from Meiji Dairies Co., Ltd. (Tokyo, Japan). Marigold pigment (0.5 mg/mL) was dissolved with a vehicle emulsion containing 0.1 mg of lecithin (Wako Pure Chemicals, Osaka, Japan) in 1 mL of water or cow milk using a supersonic wave crusher (150 W for 90 s; SONICATOR 5202, Ohtake Seisakusho, Aichi, Japan).

### Study Design

Following a 7-day acclimatization period, rats of both the sedentary and exercise groups were sub-divided into four groups by matched body weight or running distance matched with body weight: control (vehicle emulsion), lutein emulsion, cow milk, and lutein-fortified milk groups, each consisting of six rats. Running distance was matched according to the mean total running distance in the last 4 days of the acclimatization period. The period of access to the wheel-running cage was from 17∶00 to 09∶00 h, resulting in an 8-h exercise deprivation during the light period of each day. Furthermore, the rats were fasted from 13∶00 to 17∶00 h throughout the experimental period; a 4-h food deprivation occurred before administering the control or test emulsion. Rats in each group received 10 mL/kg body weight of test emulsion (with or without lutein contents; 1 mg/kg body weight) via a feeding tube (SF-FT0380FG, Fr. 3.5, o.d. 1.2 mm, Terumo, Tokyo, Japan) at 17∶00 h daily. The regular diet was made available immediately after administration of the test emulsion. Body weight, food intake, and running distances were then measured each day. On the final day of the 9-week experimental period, all rats were anesthetized with sodium pentobarbital, the abdominal cavity was opened, abdominal aortic blood was collected, and the rats were euthanized. Serum was obtained by centrifugation (1,000× *g*, 10 min, 4°C) and stored at –80°C until carotenoid concentration analysis. The liver, abdominal (mesenteric, perirenal, and epididymal) fat, and gastrocnemius muscle tissues were weighed and stored at −80°C for subsequent analyses.

### Measurement of Triglyceride and Total Cholesterol Concentrations in Serum and Liver

On day 60, 100 μL of tail blood was collected after 8 h fasting prior to administering each test emulsion. The blood samples were immediately centrifuged, and sera were stored at –80°C. Folch’s extraction method was used to assess liver triglyceride (TG) and cholesterol (CHO) levels [22]. TG and total cholesterol (T-CHO) concentrations in serum and liver were analyzed using enzymatic tests for TG and T-CHO (Wako Pure Chemical Industries, Tokyo, Japan).

### Measurement of Serum Lutein and β-carotene Concentration

Serum lutein and β-carotene concentrations were measured as previously described by Hulshof *et al*. [23]. Lutein, β-carotene, and echinenone were used as standards for the assays and obtained from CaroteNature GmbH (Lupsingen, Switzerland). Briefly, serum samples (1 mL) obtained by centrifugation from aortic blood were extracted with 250 μL of ammonium solution, 1 mL of 96% ethanol, 1 mL of diethylether in butylhydroxyltoluene, and 1 mL of petroleum ether. This solution was then added to 100 μL of echinenone (internal standard, 5 μM), and centrifuged (2 min, 9,000× *g*) and the supernatants were collected. This extraction procedure was repeated three times. The supernatants were dried and saponified in 1 mL of ethanol in potassium hydroxide solution at room temperature for 3 h. The sample solution was extracted by hexane, dried, and dissolved in 100 μL methanol-tetrahydrofuran (3∶1) solution. Lutein and β-carotene were then quantified by high-performance liquid chromatography (HPLC) (LaChromElite-DAD, Hitachi High Technologies Co., Tokyo, Japan). The HPLC system was fitted with a 3-μm Imtakt Cadenza CD-C18 column (150 × 2 mm; Imtakt Co., Ltd., Kyoto, Japan), and the temperature was maintained with the column oven set at 20°C. Absorbance was monitored at 450 nm using a photodiode array detector. Solvent A (methanol:water:tetrahydrofuran:tetraethylamine, 87.9∶ 10: 2.0∶ 0.1) and B (methanol:tetrahydrofuran:tetraethylamine, 92.4∶ 7.5∶ 0.1) were run at a flow rate of 1.0 mL/min using a linear gradient from 0% to 100% for 0.2 min, held at 100% for solvent B for the next 35 min, and then returned to the initial conditions. The concentrations of lutein and β-carotene were calculated using the calibration curves measured from standard solutions.

### SDS-PAGE and Western Blotting

Gastrocnemius muscle was homogenized in lysis buffer (CelLytic MT, Sigma-Aldrich, St. Louis, MO, USA) containing phosphatase and protease inhibitors (Phosphatase Inhibitor Cocktail, Nakalai Tesuque, Inc., Kyoto, Japan) using a Biomasher (Micro Smash MS-100, Tomy Seiko Co., Ltd., Tokyo, Japan). The cell debris of the homogenates was removed by centrifugation (15,000× *g*, 10 min) and total protein contents were quantified using a protein assay (Bio-Rad Laboratories, Inc., Tokyo, Japan). To analyze protein expression [24], 20 μg of protein lysates were resolved by sodium dodecyl sulphate polyacrylamide gel electrophoresis (SDS-PAGE, 4–12%) and transferred to a polyvinylidene difluoride (PVDF) membrane using a semi-dry transfer apparatus (iBlot, Invitrogen Co, Carlsbad, CA, USA). Membranes were blocked with 5% non-fat dry milk (Cell Signaling Technology, Inc., Danvers, MA, USA) in Tris-buffered saline with 0.1% Tween 20, and probed with the appropriate primary antibodies: phosphospecific AMPK (Thr172) (Cell Signaling Technology, Inc.), total AMPK (Cell Signaling Technology, Inc.), CPT-1M (Santa Cruz Biotechnology, Inc., Santa Cruz, CA, USA), and β-Actin (Abcam, Tokyo, Japan). All Western blots were visualized using an immunoreaction enhancer solution (Can Get Signal, Toyobo Co., Ltd., Osaka, Japan). Proteins were visualized with enhanced chemiluminescence reagents (Thermo Fisher Scientific, Kanagawa, Japan). The signal was quantified with a Lumino Image Analyzer LAS-1000 System (Fuji Photo Film, Tokyo, Japan).

### Statistical Analyses

All values are expressed as means ± standard error of the mean (SEM). The effects of exercise, milk, or lutein administration on the levels of TG and T-CHO in the liver and gastrocnemius were analyzed using repeated-measures one-way or a three-way analysis of variance (ANOVA). Differences in running distances among the groups were analyzed with a one-way ANOVA. When the ANOVA showed a statistically significant main effect, pair-wise comparisons were performed using Tukey–Kramer’s HSD test to determine differences between means. Differences at *P*<0.05 were considered statistically significant. Effect size values were calculated for the exercise, milk, and lutein factors, and the 95% confidence interval was reported [25]. All data were analyzed using the JMP software (ver. 8.1; SAS Institute Japan, Tokyo, Japan).

## Results

### Body Weight Gain, Food Intake, Total Weight of Abdominal Fat Tissue and Gastrocnemius Muscle Weight

The initial and final body weights did not differ among the groups (Table 1). Total food intake was significantly higher in the exercise group than in the sedentary groups (Exercise; *p*<0.001, three-way ANOVA); however, no significant difference was observed between milk and lutein-treated rats (Milk; *p = *0.377, Lutein; *p = *0.316, three-way ANOVA). The total weight of abdominal fat tissue was significantly lower in the exercise and milk-treated groups compared with the remaining groups (Exercise; *p*<0.001, Milk; *p = *0.031, three-way ANOVA). The weight of gastrocnemius muscle in exercise and milk-treated groups was elevated (Exercise; *p*<0.001, Milk; *p = *0.033, three-way ANOVA).

**Table 1:** Final body weight, total food intake, abdominal fat, and gastrocnemius weight.

		Final body weight	Total food intake	Abdominal fat	Gastrocnemius
		g	g/100 g body weight		
Sedentary	Control	274.5±2.2	797.5±5.9^bc^	7.99±0.20^a^	0.52±0.004^a^
	Lutein	282.0±4.48	811.2±15.0^ab^	7.69±0.28^a^	0.52±0.006^a^
	Milk	285.3±4.1	786.2±12.9^c^	7.29±0.18^a^	0.55±0.009^ab^
	Milk+Lutein	279.7±3.3	791.4±11.3^c^	7.90±0.08^a^	0.53±0.009^a^
Exercise	Control	279.9±4.8	852.2±5.3^ab^	4.80±0.26^b^	0.59±0.005^bc^
	Lutein	292.9±7.0	859.6±13.7^a^	5.24±0.39^b^	0.57±0.019^b^
	Milk	279.6±8.2	848.3±19.3^ab^	4.59±0.35^b^	0.61±0.019^c^
	Milk+Lutein	284.2±6.0	869.8±13.9^a^	4.07±0.45^b^	0.57±0.009^bc^
Three-way ANOVA *P*-values	Exercise	0.325	<0.001	<0.001	<0.001
	Milk	0.978	0.377	0.031	0.033
	Lutein	0.206	0.316	0.781	0.024
	Exercise+Milk	0.254	0.389	0.29	0.585
	Exercise+Lutein	0.304	0.959	0.647	0.171
	Milk+Lutein	0.21	0.857	0.948	0.21
Effect Size (95% CI)	Exercise	0.29 (−0.29 to 0.85)	2.07 (1.34 to 2.73)	−3.96 (−4.86 to −2.93)	2.12 (1.38 to 2.79)
	Milk	−0.01 (−0.57 to 0.56)	−0.22 (−0.78 to 0.35)	−0.27 (−0.84 to 0.30)	0.41 (−0.17 to 0.97)
	Lutein	0.38 (−0.20 to 0.94)	0.27 (−0.30 to 0.84)	0.03 (−0.53 to 0.60)	−0.43 (−1.00 to 0.15)

Weights of total abdominal fat and the gastrocnemius muscle following exercise and oral feeding treatment at day 63 from the start of the test period are shown. Values are expressed as means ± SEM (*n = *6). Statistical analyses were performed with one-way and three-way analyses of variance (ANOVAs). A *p*-value <0.05 among the eight groups by the Tukey–Kramer test was deemed to indicate statistical significance. Values with different letters were significantly different among the eight groups according to exercise condition.

### Effects of Milk and Lutein on Running Exercise

**Figure 1:**
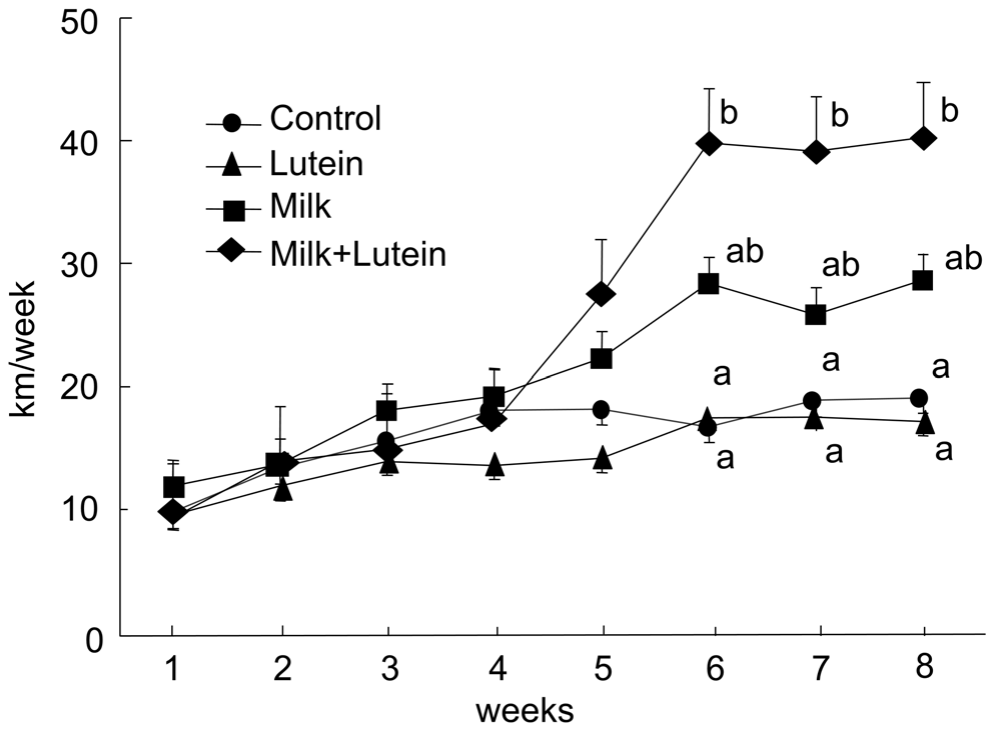
Running distances. Changes in the total running distances per week during the test period. Rats in each group were administrated 10/kg orally of each test emulsion prior to onset of the dark period of the light/dark cycle. Running distances were measured during the dark period between 17∶00 and 5∶00 h daily. Values are expressed as means ± SEM (*n = *6). Statistical analyses were performed using a one-way analysis of variance. A *p*-value <0.05 by the Tukey–Kramer test among the eight groups was deemed to indicate statistical significance. Values with different letters show significant differences among groups at each time point.

The total weekly running distances of the lutein-fortified milk group increased significantly from 6 weeks to the end of the test period, whereas weekly running distances did not change in the lutein-administered alone group (Fig. 1). The milk-administered group showed a tendency to increase the total weekly running distance. Although, in the lutein-fortified milk group, daily running distance also increased significantly at week 8, similar to total weekly running distance, this increase was not observed with the administration of lutein alone (data not shown).

### Concentrations of TG and T-CHO in Blood and Liver

The fasting blood concentrations of TG and T-CHO in the exercise and milk-treated groups were lower than those in the other groups (Table 2, TG: Exercise; *p = *0.001, Milk; *p = *0.022, T-CHO: Exercise; *p*<0.001, Milk; *p = *0.019, three-way ANOVA). The TG and T-CHO contents in the liver were also significantly reduced by both exercise and milk treatment (Table 2, TG: Exercise; *p*<0.001, Milk; *p = *0.013, T-CHO: Exercise; *p*<0.001, Milk; *p = *0.018, three-way ANOVA).

**Table 2:** Concentrations of triglyceride (TG) and total cholesterol (T-CHO) in blood and liver.

		Blood (mmol/L)			Liver (mmol/g)				
		Triglyceride	Total cholesterol		Triglyceride		Total cholesterol		
Sedentary	Control	1.44±0.21^ab^	2.71±0.16^a^		23.8±2.12^a^		7.68±0.57^a^		
	Lutein	1.56±0.14^a^	2.53±0.33^a^		22.7±1.76^a^		7.68±0.14^a^		
	Milk	1.13±0.14^ab^	2.61±0.31^a^		19.0±1.82^ab^		6.97±0.47^ab^		
	Milk+Lutein	1.35±0.14^ab^	2.53±0.16^a^		18.6±1.50^ab^		6.82±0.44^ab^		
Exercise	Control	1.11±0.14^ab^	2.24±0.17^ab^		17.5±1.76^ab^		6.63±0.40^ab^		
	Lutein	1.12±0.1^ab^	2.28±0.24^ab^		16.6±1.81^ab^		6.19±0.29^ab^		
	Milk	0.80±0.20^b^	1.94±0.35^b^		14.3±0.98^b^		5.85±0.49^ab^		
	Milk+Lutein	0.85±0.12^ab^	1.88±0.16^b^		12.5±1.26^b^		5.38±0.62^b^		
Three-way ANOVA *P*-values	Exercise	0.001		<0.001		<0.001		<0.001	
	Milk	0.022		0.019		0.013		0.018	
	Lutein	0.392		0.393		0.387		0.413	
	Exercise+Milk	0.907		0.081		0.724		0.974	
	Exercise+Lutein	0.557		0.464		0.826		0.562	
	Milk+Lutein	0.755		0.973		0.977		0.924	
Effect Size (95% CI)	Exercise	−1.01 (−1.59 to −0.39)		−1.06 (−1.64 to −0.44)		−1.32 (−1.93 to −0.68)		−1.14 (−1.73 to −0.51)	
	Milk	−0.63 (−1.20 to −0.04)		−0.32 (−0.88 to 0.26)		−0.84 (−1.41 to −0.23)		−0.64 (−1.21 to −0.05)	
	Lutein	0.22 (−0.35 to 0.78)		−0.02 (−0.58 to 0.55)		−0.20 (−0.76 to 0.37)		−0.21 (−0.77 to 0.37)	

Concentrations of TG and T-CHO in fasting blood and liver in response to exercise and oral feeding treatment at days 60 and 63, respectively. Values are expressed as means ± SEM (*n = *6). Statistical analyses were performed using one-way and three-way analyses of variance (ANOVAs). A *p*-value <0.05 among the eight groups by the Tukey–Kramer test was deemed to indicate statistical significance. Different letters indicate significant differences among the eight groups according to exercise condition.

### Serum Lutein and β-carotene Concentration

The serum lutein concentration was significantly higher in the lutein-fortified milk-treated group than in the lutein alone-treated groups (Table 3, Tukey–Kramer, *p*<0.05, one-way ANOVA). The level of lutein in blood was significantly decreased by exercise, whereas it was increased by milk administration (Exercise; *p = *0.010, Milk; *p*<0.001, three-way ANOVA). Lutein was undetectable in the serum of the control and milk groups. No significant difference was observed among the groups when levels of β-carotene were assessed.

**Table 3:** Serum lutein and β-carotene concentrations.

		Lutein	β-carotene	
		nmol/L		
Sedentary	Control	–^c^	2.95±0.21	
	Lutein	12.1±1.53^b^	3.08±0.46	
	Milk	–^c^	3.01±0.33	
	Milk+Lutein	34.1±6.95^a^	3.46±0.53	
Exercise	Control	–^c^	2.39±0.66	
	Lutein	9.20±1.26^bc^	2.81±0.50	
	Milk	–^c^	2.80±0.36	
	Milk+Lutein	15.7±1.29^a^	3.42±0.40	
Three-way ANOVA *P*-values	Exercise	0.01		0.301
	Milk	<0.001		0.275
	Lutein	<0.001		0.248
	Exercise+Milk	0.048		0.311
	Exercise+Lutein	0.007		0.941
	Milk+Lutein	<0.001		0.595
Effect Size (95% CI)	Exercise	−0.42 (−0.99 to 0.16)		−0.20 (−0.76 to 0.37)
	Milk	0.58 (−0.01 to 1.14)		0.29 (−0.28 to 0.86)
	Lutein	1.93 (1.22 to 2.59)		0.34 (−0.24 to 0.90)

The concentrations of serum lutein and β-carotene in response to exercise and oral feeding at day 63. Values are expressed as means ± SEM (*n = *6). Statistical analyses were performed using one-way and three-way analyses of variance (ANOVAs). A *p*-value <0.05 among the eight groups by the Tukey–Kramer test was deemed to indicate statistical significance. Different letters indicate significant differences among the eight groups according to exercise condition.

### CPT-1 and AMPK Contents in Gastrocnemius Muscle by Western Blotting

**Figure 2:**
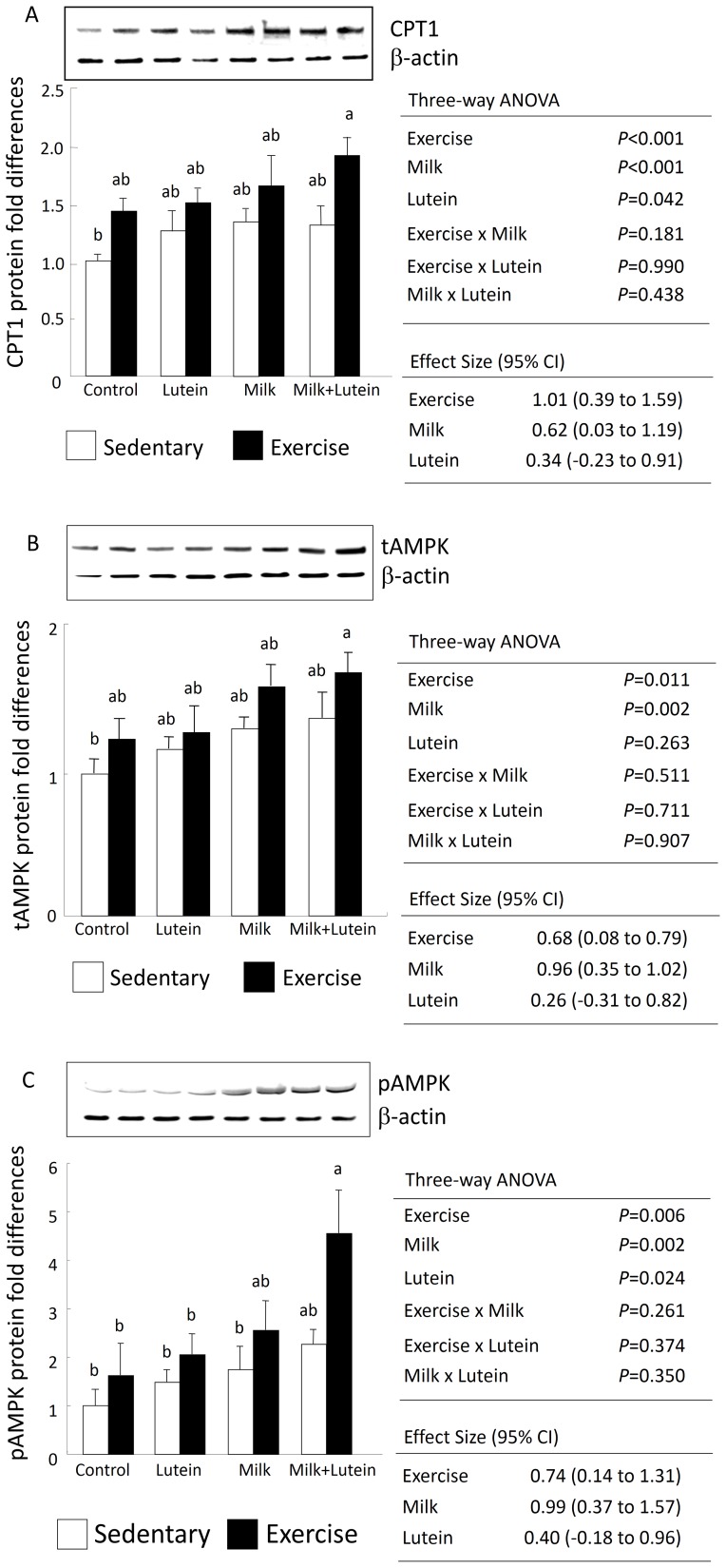
Protein expression of lipid-metabolizing enzymes in the gastrocnemius muscle following exercise and oral feeding. Cell lysates were resolved by SDS-PAGE and probed for (A) CPT-1, (B) total-AMPK, and (C) phosphorylated-AMPK antibodies as described in the Methods. CPT-1, total-AMPK, and phosphorylated-AMPK levels were normalized by β-actin and are expressed as relative values normalized to the sedentary control. Values are expressed as means ± SEM (*n = *6). Statistical analyses were performed using one-way and three-way analyses of variance (ANOVAs). A *p*-value <0.05 by the Tukey–Kramer test among the eight groups was deemed to indicate statistical significance. Different letters indicate significant differences among the eight groups according to exercise condition.

When the levels of CPT-1, total AMPK (tAMPK), and phosphorylated AMPK (pAMPK; AMPK is phosphorylated on its α-subunit at Thr172 when activated) were assessed, significantly higher levels were observed in both the exercise and milk-treated groups compared with the sedentary and non-milk-treated groups (Fig. 2A–C, CPT-1: Exercise; *p*<0.001, Milk; *p*<0.001, tAMPK: Exercise; *p = *0.011, Milk; *p = *0.002, pAMPK: Exercise; *p = *0.006, Milk; *p = *0.002, three-way ANOVA). Additionally, the levels of CPT-1 and pAMPK were significantly higher in the lutein-treated groups than in the non-lutein-treated groups (Fig. 2A and C, CPT-1: Lutein; *p = *0.042, pAMPK: Lutein; *p = *0.024, three-way ANOVA). Furthermore, the pAMPK levels were four-fold higher in the exercise and milk with lutein-treated rats compared with the sedentary control group (Fig. 2C, Tukey–Kramer, *p*<0.05, one-way ANOVA).

## Discussion

In the current study, we demonstrated that wheel-running exercise increased total food intake and gastrocnemius weight, decreased abdominal fat, and reduced levels of blood and liver TG and T-CHO (Table 1 and 2). These data indicate that lipid metabolism is enhanced, likely due to the elevated energy consumption associated with running exercise [26]. Furthermore, we observed that lutein-fortified milk increased the weekly running distance of wheel-running rats after week 6 of the test period (Fig. 1). Thus, these results indicate that supplementation of lutein-fortified milk enhances the effect of exercise, with an improvement in lipid metabolism.

A previous study found that exercise *per se* induced the expression of CPT-1 and AMPK [27]. We observed a two-fold increase in CPT-1 content in the lutein-fortified milk- and exercise-treated groups compared with the sedentary control group. Aoi *et al*. [28] reported that astaxanthin, a marine carotenoid, improved muscle lipid metabolism in exercise via an inhibitory effect of oxidative CPT-1 modification in the gastrocnemius muscle of treadmill-treated rats. This mechanism may be related to the effect of lutein fortified-milk on cellular CPT-1 levels.

AMPK functions as a sensor of the intracellular energy state and is activated by exercise, adiponectin, leptin, and sympathetic outflow in skeletal muscle [29]. Treatment with AMPK agonists has been found to improve lipid metabolism and enhance exercise endurance in mice [9]. Our findings demonstrated that voluntary exercise and milk administration independently increased the expression of AMPK protein in the gastrocnemius muscle. Furthermore, AMPK activation (measured by means of an elevation of Thr172 phosphorylation) was strongly enhanced by lutein supplementation of milk. Thus, administration of milk in the presence of lutein may lead to increased total running distance through increased energy metabolism mediated by AMPK activation. Systematic training is thought to provide a higher level of activity and intensity than voluntary exercise [30]; thus, a treadmill or swimming exercise protocol may have produced a clearer exercise result and more marked effect of lutein.

Antioxidants are thought to ameliorate oxidative stress and promote the use of fatty acids in the mitochondria during aerobic exercise [27]. However, although vitamins C and E have anti-oxidative activity [31], [32], the presence or absence of such an effect of using fatty acid is not clear. Moreover, the relationship between improved utilization of fatty acid and increased endurance is not well understood. Thus, although the effect of antioxidants on exercise is not well understood, the increased running distance associated with administration of lutein-fortified milk shown in our results may be the result of antioxidant properties. The decrease in blood concentrations of β-carotene caused by oxidative damage related to vigorous exercise is thought to protect against exercise-induced stress [33]. Furthermore, we found that running exercise decreased circulating lutein levels with no change in the concentration of β-carotene, suggesting that lutein may save the consumption of other antioxidant components. Considering that oxidative stress induction accompanies increased exercise levels, it is difficult to identify the precise inhibitory effects against oxidative stress. Thus, the anti-oxidative property of lutein requires further investigation.

We found that lutein administration alone had no effect on running distances, but in combination with milk, total running distances increased (Fig. 1). By combining the intake of lutein with dairy products, the amount of lutein absorbed would be expected to be enhanced [13]. Accordingly, serum lutein concentrations in our study doubled when lutein was combined with milk. Lutein treatment combined with quercetin (a plant-derived flavonoid) had no effect on oxidative stress status or on plasma concentration of lutein in adequately nourished older adults [34], indicating that lutein administration alone leads to poor absorption. This may explain why a previous study failed to show an effect of quercetin on exercise endurance in male adults [35]. Similarly, the load or intensity of the voluntary exercise in our study may have been insufficient to produce a lutein-induced response. Thus, future studies that use high-intensity exercise, such as the treadmill, and assess plasma lutein levels under strenuous exercise conditions or studies that deliver a single high dose of lutein comparable to the plasma levels of lutein-fortified milk administration, may be required to explain why lutein administration alone had no effect on running distances.

There may be other reasons for increasing voluntary running distance in a combination of cow’s milk and lutein. We guess that in addition to promoting the absorption of lutein and increasing plasma levels of the nutrient, the protein composition of milk may improve glucose or lipid metabolism and increase the amount of physical activity as an effect of milk absorption itself [36], [37]. In fact, the weight of the gastrocnemius muscle in the milk alone group increased more than that of the lutein alone group. Taken together, these findings suggest that milk enhances lutein absorption, and simultaneous intake improved muscle glucose or lipid metabolism and physical performance. That is, the effect of lutein on exercise performance relies on its being ingested with milk. Thus, lutein may not show a simple dose–response effect.

Lutein improves cognitive function in ageing animals [18] and people by combining with docosahexaenoic acid [38]. Moreover, in previous study, we showed that voluntary exercise was increased in mice fed the breast milk of mothers that had consumed lutein [39]. This finding may have been associated with the improved cognitive function and an increase in the effect of exercise. However, it is difficult to draw this conclusion from our results because we did not assess changes in mood or cognitive function; thus, further study is needed to clarify this point. Regarding the short-term effect of exercise, we observed that milk administration significantly increased running distance during the initial hour of exercise, and lutein-fortified milk maintained this increase beyond the initial hour (data not shown), suggesting a synergistic effect of milk and lutein on these processes. These increases in running distances were not observed when the rats were treated with lutein alone. Although further studies are required, at present, there are insufficient reports of the components derived from foods that focused on the relationship between cognitive function improvement and an increase in the effect of exercise.

In conclusion, exercise and milk intervention improved lipid metabolism synergistically. Furthermore, lutein-fortified milk enhanced the effect of exercise due to improved lipid metabolism through milk administration and the possible anti-oxidative properties of lutein. The current study may provide a novel market for the potential inclusion of antioxidants in milk, allowing improvements in lipid metabolism. Further clarification is now required to assess the translocation of these data into human adult subjects ranging from athletes to obese individuals.
